# FRET-Based Detection of M_1_ Muscarinic Acetylcholine Receptor Activation by Orthosteric and Allosteric Agonists

**DOI:** 10.1371/journal.pone.0029946

**Published:** 2012-01-17

**Authors:** Danijela Markovic, Jonathan Holdich, Suleiman Al-Sabah, Rajendra Mistry, Cornelius Krasel, Martyn P. Mahaut-Smith, R. A. John Challiss

**Affiliations:** 1 Department of Cell Physiology and Pharmacology, University of Leicester, Leicester, United Kingdom; 2 Department of Physiology, Development & Neuroscience, University of Cambridge, Cambridge, United Kingdom; 3 School of Pharmacy, University of Reading, Whiteknights, Reading, United Kingdom; Cornell University, United States of America

## Abstract

**Background and Objective:**

Muscarinic acetylcholine receptors (mAChRs) are 7-transmembrane, G protein-coupled receptors that regulate a variety of physiological processes and represent potentially important targets for therapeutic intervention. mAChRs can be stimulated by full and partial orthosteric and allosteric agonists, however the relative abilities of such ligands to induce conformational changes in the receptor remain unclear. To gain further insight into the actions of mAChR agonists, we have developed a fluorescently tagged M_1_ mAChR that reports ligand-induced conformational changes in real-time by changes in Förster resonance energy transfer (FRET).

**Methods:**

Variants of CFP and YFP were inserted into the third intracellular loop and at the end of the *C*-terminus of the mouse M_1_ mAChR, respectively. The optimized FRET receptor construct (M_1_-cam5) was expressed stably in HEK293 cells.

**Results:**

The variant CFP/YFP-receptor chimera expressed predominantly at the plasma membrane of HEK293 cells and displayed ligand-binding affinities comparable with those of the wild-type receptor. It also retained an ability to interact with Gα_q/11_ proteins and to stimulate phosphoinositide turnover, ERK1/2 phosphorylation and undergo agonist-dependent internalization. Addition of the full agonist methacholine caused a reversible decrease in M_1_ FRET (F_EYFP_/F_ECFP_) that was prevented by atropine pre-addition and showed concentration-dependent amplitude and kinetics. Partial orthosteric agonists, arecoline and pilocarpine, as well as allosteric agonists, AC-42 and 77-LH-28-1, also caused atropine-sensitive decreases in the FRET signal, which were smaller in amplitude and significantly slower in onset compared to those evoked by methacholine.

**Conclusion:**

The M_1_ FRET-based receptor chimera reports that allosteric and orthosteric agonists induce similar conformational changes in the third intracellular loop and/or *C*-terminus, and should prove to be a valuable molecular reagent for pharmacological and structural investigations of M_1_ mAChR activation.

## Introduction

Muscarinic acetylcholine receptors (mAChRs) are 7-transmembrane domain proteins that belong to the rhodopsin family of G protein-coupled receptors (GPCRs). mAChRs are widely distributed and are responsible for the metabotropic effects of acetylcholine. mAChR subtypes are encoded by 5 distinct genes in mammals, referred to as M_1_–M_5_. M_2_ and M_4_ mAChR subtypes couple predominantly through pertussis toxin-sensitive G_i/o_ proteins to inhibit adenylyl cyclase, whereas M_1_, M_3_ and M_5_ mAChR subtypes preferentially couple via pertussis toxin-insensitive G_q/11_ proteins to activate phospholipase C-β, mobilize intracellular Ca^2+^, regulate protein kinase C, and modulate a variety of Ca^2+^ and K^+^-channels [1], [2].

mAChRs have been implicated in both the aetiology and potential treatment of a number of psychiatric and neurological conditions, including schizophrenia and Alzheimer's disease [3], [4]. Despite substantial efforts over a period of 50 years to develop chemicals that can pharmacologically target specific mAChR subtypes, it is only very recently that truly subtype-selective ligands have been reported [5], [6]. The majority of the newly reported compounds appear to interact with the receptor at sites distinct from the orthosteric binding pocket, which has been shown to be highly conserved across the M_1_–M_5_ mAChRs [7], [8]. Thus, binding and activation of M_1_–M_5_ mAChRs by orthosteric ligands is mediated largely though interactions with a small number of key residues (e.g. Y^381^ and Q^382^ in TM6 (numbering refers to M_1_ receptor) [9]), leading to a relative change in the orientation of TM3 and TM6 of the receptor and a consequent change in the distance between the *C*-terminus and third intracellular (i3) loop [10].

That mAChRs can bind ligands at a variety of non-orthosteric sites is now well documented [5], [6]. Of particular interest is a structurally diverse group of compounds that achieve functionally selective agonism at M_1_ mAChRs though interactions at receptor binding sites topologically distinct from the acetylcholine binding pocket [11]–[13]. With respect to AC-42 [14] and 77-LH-28-1 [12], Lebon and colleagues have proposed a novel “conformational trapping” mechanism for activation of the M_1_ mAChR by these ligands [15]; in contrast, *N*-desmethylclozapine, another allosterically-acting agonist, and the natural ligand acetylcholine do not employ the same conformational trapping mechanisms to activate the receptor.

In the present study we have investigated ligand-induced conformational changes of M_1_ mAChRs using Förster resonance energy transfer (FRET). The binding of an agonist to a GPCR results in conformational changes to the protein, including changes in the relative distance between the third intracellular loop and the *C*-terminus of the receptor. This movement can be monitored in real-time in live cells using FRET between CFP and YFP, genetically modified variants of green fluorescent protein (GFP) as introduced by Lohse and colleagues [16], [17]. While the CFP/YFP-GPCR chimera is believed to report wild-type GPCR conformational change upon agonist binding, its ability to link to G proteins is often abolished. Here, we have developed a M_1_-FRET biosensor that retains an ability to signal through its G_q/11_-coupled pathway, and have used this to report conformational change on binding of allosteric and orthosteric agonists.

## Materials and Methods

### Materials

Dulbecco's modified Eagle's medium with GlutaMAX (DMEM), penicillin-streptomycin (pen/strep), G418, fetal bovine serum (FBS), Lipofectamine™ 2000, restriction enzyme, pcDNA3 and competent *E.coli* (top tens) were purchased from Invitrogen (Paisley, UK). The source of EYFP and ECFP was pEYFP-C1 and pECFP-C1, respectively (Clontech, California, USA). PCR chemicals were obtained from Promega (Southampton, UK). 77-LH-28-1 and AC-42 were kind gifts from GlaxoSmithKline (Harlow, UK). All other chemicals were purchased from Sigma-Aldrich unless otherwise stated.

### Modification of pECFP-C1 and pEYFP-C1

M_1_ mAChRs were tagged with the cerulean mutant of ECFP (ECFPc) [18], which required three point mutations (S72A/Y145A/H148D) and an improved version of EYFP, with a single point mutation (F46L) that greatly enhances its fluorescence (referred to as EYFP^F46L^) [19]. Mutagenesis was performed using the QuikChange point-directed mutagenesis kit (Stratagene, California, USA). The vectors generated are referred to as pECFPc-C1 and pEYFP^F46L^-C1, respectively.

### Isolation of murine M_1_ mAChR and labelling at the C-terminus with EYFP^F46L^

The care and use of animals in this study was in accordance with the UK Animals (Scientific Procedures) Act 1986 and authorised by the University of Cambridge certificate of designation (reference no. PCD 80/2802). The investigation also conforms to the Guide for Care and Use of Laboratory Animals US (NIH Publication No. 85-23, revised 1996). Male mice aged between 10 and 24 weeks old were killed by cervical dislocation. The brain was removed and immediately frozen with liquid nitrogen and ground to a fine powder in a mortar and pestle under liquid nitrogen. Total RNA extraction was carried out using a Qiagen RNeasy mini-kit following the manufacturer's instructions. The required amount of tissue was re-suspended in the accompanying lysis buffer, containing β-mercaptoethanol (final concentration 143 mM) and was homogenized using a glass hand-held homogenizer. The lysate was then passed 10 times through a 21G syringe needle. cDNA was generated from total RNA using a reverse transcription kit, Omniscript (Qiagen, Crawley, UK). The 50 µL reaction contained: 1× RT buffer, 0.5 mM dNTPs, 25 ng/µL Oligo-dT and random hexamer primers, 0.5 U/µL Rnasin, 0.2 U/µL Omniscript reverse transcriptase, 2.5 µg total RNA, Rnase free water to final volume. The mixture was incubated at 37°C for 1 h. PCR from a mouse cDNA template was used to generate the full-length M_1_ mAChR DNA minus the stop codon for insertion into a plasmid. The 50 µL PCR reaction included: 1× Thermopol buffer, 0.2 mM dNTPs (Bioline Ltd, London, UK), 0.5 µM forward primer 5′ATGAACACCTCAGTGCCCCCTGC3′, 0.5 µM reverse primer 5′TTAGCATTGGCGGGAGGGGGTGC3′, 0.5 U Vent polymerase and UV-treated milliQ water to 50 µL. Amplification was carried out using a Mastercycle gradient thermocycler (Eppendorf UK Ltd., Cambs, UK). The PCR employed an initial denaturation step of 95°C for 3 min followed by 35 cycles of 95°C for 30 sec denaturation, 69°C for 30 sec annealing, 72°C for 2 min extension, and a final single 72°C for 10 min extension then held at 4°C. The product from this PCR was cleaned and used as a PCR template to which *Bam HI* and *EcoR I* sticky ends were added. The PCR mixture was as above but with forward primer 5′ATACGGATCCATGAACACCTCAGTGCCC3′ and reverse primer 5′GTATGAATTCAAGCATTGGCGGGAGGGGG3′. The product was cleaned, digested sequentially with *BamHI* and *EcoRI* and ligated into pEYFP^F46L^-N1. This gave a construct that would express as a murine M_1_ mAChR to which EYFP^F46L^ was attached via a 6 residue linker (LNSADI) after the terminal C^460^. This construct was named M_1_-YFP^CT^.

### Addition of ECFPc to the third intracellular loop of the M_1_ mAChR

Using point directed mutagenesis, an *Age I* restriction site (ACCGGT) was added to the third intracellular loop of M_1_-YFP^CT^ in 5 different positions (see Table 1). The modified vectors produced by mutagenesis were digested overnight with *Age I*. The digested plasmids were run on a 1% agarose gel to remove uncut vector cleaned. Using PCR with peCFPc-N1 as a template and primers forward 5′ATACACCGGTATGGTGAGCAAGGGCGAGG3′ and reverse 5′GTATACCGGTCTTGTACAGCTCGTCCATGC3′, *Age I* restriction sites were added to the ends of eCFPc. The PCR product was cleaned, digested overnight with *Age I* and ligated into the cut vector with Quick Ligase (NEB, Herts, UK) by incubation for 20 min at room temperature (molar ratio of insert to vector of 5∶1). The ligated material was used to directly transform competent *E.coli* (top tens) according to the manufacturer's protocol. The resultant plasmids were checked for insert and sequenced. The summary of created constructs is presented in Table 1. For control purposes, constructs equivalent to M_1_-cam5, but containing only *C*-terminal EYFP^F46L^ or ECFPc in the third intracellular loop, were also created. These constructs are referred to as M_1_-cterm-EYFP^F46L^ and M_1_-ic3-ECFPc, respectively.

**Table 1:** Overview of M_1_ AChR chimeric constructs.

Construct	Position of ECFPc	Primers
**M_1_-cam1**	Between G^340^ and Q^341^	Forward: CGAGGCGGCAAAGGCACCGGTCAAAAACCCCGAGGGReverse: CCCTCGGGGTTTTTGACCGGTGCCTTTGCCGCCTCG
**M_1_-cam2**	Between P^323^ and N^324^	Forward: CCCAAAAGCTCCCCAACCGGTAATACAGTCAAGAGGCCReverse: GGCCTCTTGACTGTATTACCGGTTGGGGAGCTTTTGGG
**M_1_-cam3**	Between P^252^ and N^324^	Forward: GCTGAAGGCTCACCCACCGGTAATACAGTCAAGAGGReverse: CCTCTTGACTGTATTACCGGTGGGTGAGCCTTCAGC
**M_1_-cam4**	Between E^242^ and K^362^	Forward: AGCAGCAGCTCTGAGACCGGTAAGGCAGCTCGGACCReverse: GGTCCGAGCTGCCTTACCGGCTCTTGAGCTGCTGCT
**M_1_-cam5**	Between K^361^ and K^362^	Forward: CTGGTCAAGGAGAGACCGGTAAGGCAGCTCGGACCReverse: GGTCCGAGCTGCCTTACCGGTCTTCTCCTTGACCAG
**M_1_-ic3-ECFPc**	Between K^361^ and K^362^	Forward: CTGGTCAAGGAGAGACCGGTAAGGCAGCTCGGACCReverse: GGTCCGAGCTGCCTTACCGGTCTTCTCCTTGACCAG

Primers used for introducing *AgeI* site into the i3 loop are shown where applicable.

### HEK293 cell culture and transfection with M_1_ mAChR constructs

Complementary DNA of chimeric receptors were transiently expressed in Human embryonic kidney 293 (HEK293) cells (ECACC Cat no. 85120602) using the Lipofectamine™ 2000 (Invitrogen) according to manufacturer's instructions. For generation of HEK293 cell-line stably expressing M_1_-cam5 mAChR, the plasmid was transfected using Lipofectamine™ 2000 reagent (Invitrogen). The cells were grown in DMEM in the presence of 500 µg/mL G418 and those that survived were sub-cultured. The newly established cell-line was termed HEK293-M_1_-cam5.

### *N*-methyl-[^3^H]scopolamine binding

*N*-methyl-[^3^H]scopolamine ([^3^H]NMS) inhibition binding assays were carried out as described previously [20]. Briefly, HEK293-M_1_-cam5 cells were seeded at a density of 75,000 cells/well in 24-well plates. After 24 h cells were washed three times with warmed KHB (composition: 118 mM NaCl, 8.5 mM HEPES, 4.7 mM KCl, 4 mM NaHCO_3_, 1.3 mM CaCl_2_, 1.2 mM MgSO_4_, 1.2 mM KH_2_PO_4_, 11.7 mM glucose, pH 7.4) followed by an incubation on ice in a total assay volume of 1 mL of ice-cold KHB containing various concentrations of agonists and approx. 0.2 nM [^3^H]NMS. After a 5 h incubation at 4°C cells were washed three times with ice-cold KHB before the addition of 0.1 M NaOH (500 µL). After cell solubilization, SafeFluor scintillation fluid was added, and samples were counted on a scintillation counter.

### Total [^3^H]inositol phosphate accumulation

HEK293-M_1_-cam5 and HEK293 cells were seeded at 100,000 cells/well in 24-well plates and incubated in fresh medium containing 2.5 µCi/mL [^3^H]inositol for 48 h. The assay was performed as previously described [20].

### ERK1/2 phosphorylation and western blotting

To determine ERK1/2 phosphorylation, the HEK293-M_1_-cam5 and HEK293 cells were grown in 12-well plates. The experiments and western blotting were performed as previously described [21]. Proteins were visualized using ECL reagent from GE Healthcare (Chalfont St. Giles, UK). Equal protein loading was confirmed using GAPDH-HPR antibody (1∶20,000) (Abcam, Cambridge, UK).

### Immunofluorescence confocal microscopy of receptor internalization

Cells were seeded on coverslips coated with 100 µg/mL poly-d-lysine in PBS. When 70–80% confluency was reached, in some instances the cells were treated with various concentrations of agonists at 37°C and fixed with 4% paraformaldehyde. After a 5 min wash with PBS, the slides were mounted with Slowfade Gold/DAPI (Invitrogen, UK). The slides were examined using an Olympus FV500 confocal microscope, ECFPc and EYFP^F46L^ were excited via the 458 nm and 515 nm line of the argon laser and emissions were then collected at 480–495 nm and 535–565 nm, respectively. Optical sections (0.5 µm) were taken, and representative sections corresponding to the middle of the cells are presented. For each treatment, between 20 and 30 individual cells in three random fields of view were selected and examined. Fluorescence intensity of specific regions of interest (longitudinal axis) was quantified by using the Measure function of Image J software developed at the National Institutes of Health (http://rsb.info.nih.gov/ij/), as previously described [21]. Briefly, relative quantification of intracellular (internalized) M1-cam5 was determined by measuring the amount of total fluorescence along the longitudinal axis corresponding to the intracellular space (average 4–18 ìm) excluding nucleus. Intracellular fluorescence in cells not treated with the agonist was considered to be basal fluorescence (assigned the value of 1). All other data are normalized to this basal fluorescence level.

### Acceptor photobleaching assessment of FRET

Photobleaching of the acceptor fluorophore to assess the level of FRET was performed using a Zeiss LSM 510 attached to an Axiovert 100 (Carl Zeiss, Welwyn Garden City, UK). Photo-bleaching was performed by repeated scanning of a selected area of the cell membrane with the 514 nm laser line at maximum intensity. ECFPc was excited at 458 nm and its emission selected using a 470–500 nm filter, while the EYFP^F46L^ emission was selected using a long pass 530 nm filter. The acquired data was analysed using either Zeiss LSM510 or LSM C4 Toolbox software (written by Dr C.J.Schwiening, University of Cambridge, UK).

### FRET measurements

FRET measurements were performed as described previously [22]. Briefly, HEK293 cells grown on coverslips were mounted on a Nikon Eclipse TE2000-S inverted microscope (Nikon) using an “Attofluor” cell chamber (Molecular Probes, Leiden, The Netherlands) and continuously superfused with HBS (150 mM NaCl, 10 mM HEPES, 10 mM glucose, 2.5 mM KCl, 4 mM CaCl_2_, 2 mM MgCl_2_, pH 7.4). Cells were observed using an oil immersion 63× lens, a polychrome V (Till Photonics, Gräfelfing, Germany) for excitation, and a dual emission photometric system. In order to minimize photobleaching, illumination time was set to 10–40 ms, applied with a frequency of 10 Hz. Fluorescence was measured at 535±15 nm (F_535_) and 480±20 nm (F_480_) (beam splitter DCLP 505 nm, Chroma Technology, Rockingham, VT, USA) on excitation at 436±10 nm (beam splitter DCLP 460 nm, Chroma Technology). The signals were detected by avalanche photodiodes and digitized using an analog/digital converter (Digidata 1322A, Axon Instruments, Union City, CA, USA) and stored on a PC using Axoscope software (Axon Instruments). The experiments were performed at room temperature.

### Data analysis

All data are expressed as mean ± SEM of at least three independent experiments. Radioligand binding data, FRET responses and agonist concentration-response curves were analyzed using Prism 5.0 (GraphPad Software Inc., San Diego, CA). A trace representative of at least three independent experiments is generally shown for FRET data.

## Results

### Design of M_1_-cam mAChR FRET conformational sensors

An overview of the M_1_-cameleon receptor constructs is given in Table 1. In all constructs EYFP^F46L^ is attached at the *C*-terminus. In the M_1_-cam1 mAChR construct, ECFPc is inserted into the i3 loop 25 residues from the predicted cytoplasmic start of the sixth transmembrane (TM6) helix. This design provides a comparable number of residues between the plasma membrane and the fluorophore for both CFP and YFP after accounting for ∼12 residues in the *C*-terminal that are predicted to generate a membrane-aligned helical (H8) region [23]. In the M_1_-cam2 mAChR, ECFPc is placed a further 17 residues distal to TM6 and most of the third intracellular loop, apart from twenty-two i3 residues immediately proximal to TM5, removed. M_1_-cam3 is similar to M_1_-cam2, but retains forty-two i3 residues below TM5. In the M_1_-cam4 construct ECFPc is positioned just 6 residues below TM6 and the majority of the i3 loop on the *N*-terminal side of ECFPc removed with 32 residues remaining proximal to TM5. This position was chosen to mimic the successful α_2A_-adrenoceptor cameleon (a receptor, like M_1_, with a relatively large (157 amino acid) i3 loop) generated by Lohse and colleagues [16], [17]. The M_1_-cam5 mAChR cameleon is similar to M_1_-cam4, but the i3 loop is retained intact, as this domain has been reported to be involved in a number of aspects of mAChR regulation, including receptor trafficking [24]–[26].

### Cellular localization of the constructs

**Figure 1:**
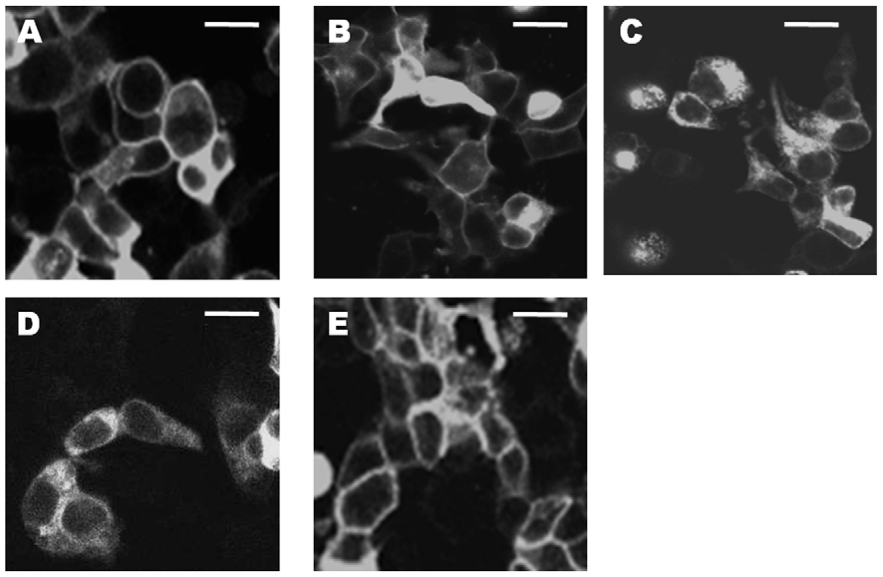
Cellular localization of M_1_-cameleons transiently expressed in HEK293 cells. HEK293 cells were transiently transfected with M_1_-cam1 (**A**), M_1_-cam2 (**B**), M_1_-cam3 (**C**), M_1_-cam4 (**D**) or M_1_-cam5 (**E**). Images were acquired by confocal microscopy and show fluorescence emission at >530 nm following excitation at 514 nm. Scale bar, 15 µm.

In order to assess the cellular localization of the cameleon receptors, HEK293 cells were transiently transfected with the appropriate cDNA. Confocal microscopy revealed that addition of the EYFP^F46L^ to the *C*-terminus of full length M_1_ mAChR did not affect plasma membrane receptor expression (data not shown). Similar results were obtained when ECFPc was inserted on its own into the i3 loop between K361 and K362 of full length M_1_ mAChR (data not shown). M_1_-cam1, -cam2, and -cam5 mAChR constructs showed good plasma membrane fluorescence with little fluorescence associated with intracellular membranes (Fig. 1A, B, E). The remaining two constructs, M_1_-cam3 and -cam4 mAChRs showed little or no plasma membrane expression (Fig. 1C, D). Removal of a large proportion of the M_1_-i3 loop in both -cam3 and -cam4 chimeras therefore compromises plasma membrane expression.

### Assessment of FRET configuration by acceptor photobleaching

**Figure 2:**
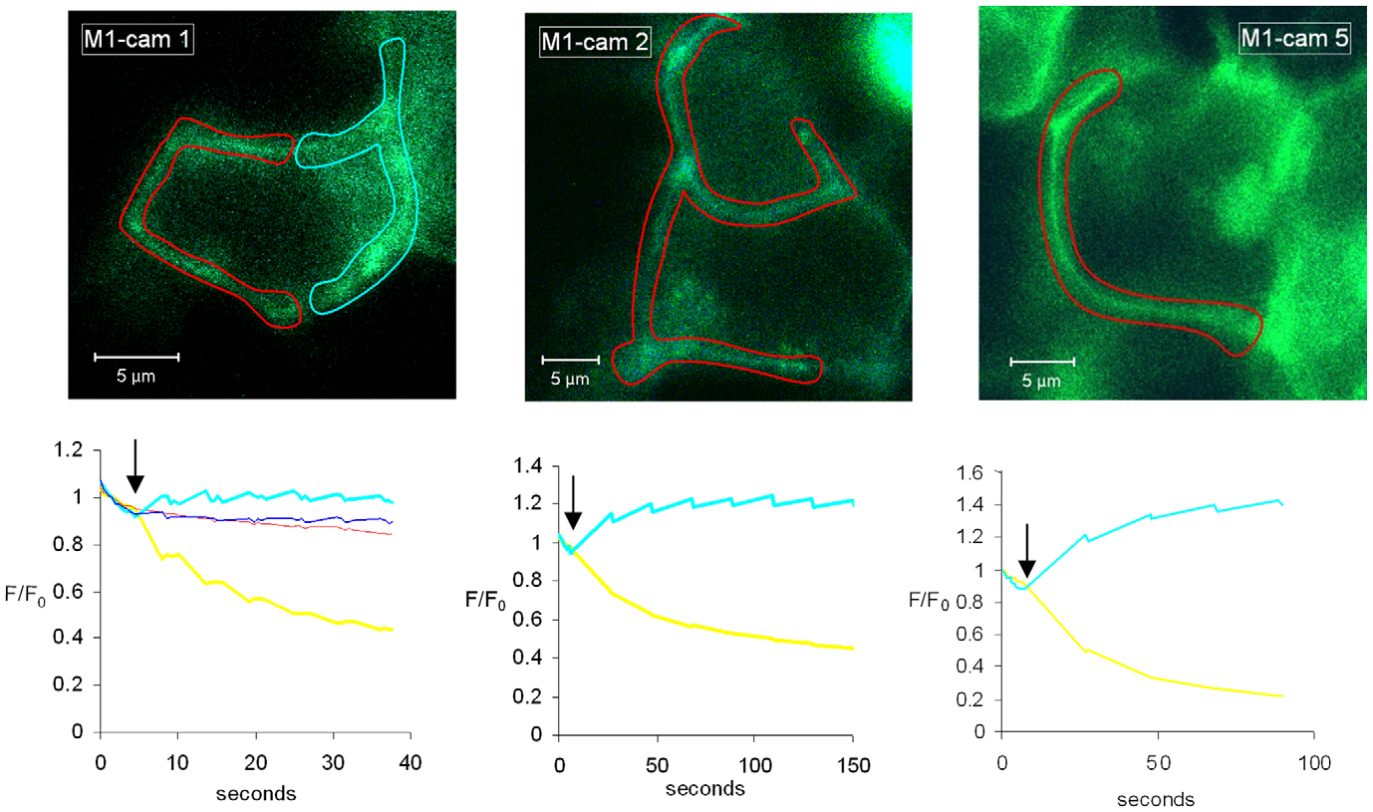
Acceptor photobleaching of the three M_1_ mAChR cameleons showing primarily plasma membrane localization. HEK293 cells were transiently transfected with M_1_-cam1, M_1_-cam2 or M_1_-cam5 and imaged by confocal microscopy (458 nm excitation, 470–500 nm emission). Areas of the plasma membrane (delineated by red lines in each image) were bleached using repeated brief exposures to high intensity 514 nm illumination. The graphs show the signal at 458 nm excitation for 470–500 nm emission (ECFPc, cyan line) and >530 nm (EYFP^F46L^ emission, yellow line), with acceptor photobleach initiated at the arrow. The fluorescence signals from a non-photobleached region were also assessed as a control, which was comparable for all constructs, but only shown for M_1_-cam1 (area outlined in cyan within the image and fluorescence within the graph for ECFPc (dark blue) and EYFP^46L^ (red)). These findings are representative of photobleaching experiments from at least 3 separate transfections for each construct.

In order to determine whether the two fluorophores are close enough to each other and in a correct orientation to generate a detectable FRET signal, we performed acceptor photobleach experiments. Only the three constructs that showed predominantly plasma membrane expression (M_1_-cam1, -cam2, and -cam5) were investigated in these experiments. All three chimeras showed an increase in donor fluorescence on photobleach of the acceptor (Fig. 2), suggesting that they all exhibit some degree of FRET under basal (ligand-free) conditions. As a control, we showed that for all three constructs a non-bleached area showed no change in either emission channel observed with illumination at 458 nm (Fig. 2, shown only for M_1_-cam1). The M_1_-cam5 receptor showed the greatest signal changes on bleaching of EYFP^F46L^, thus this construct was chosen for creation of a stable cell-line, HEK293-M_1_-cam5.

### [^3^H]NMS radioligand binding

M_1_ mAChR binding affinities for methacholine (MCh), oxotremorine-M (oxo-M), AC-42, 77-LH-28-1, arecoline, atropine and pirenzepine were determined by [^3^H]NMS competition binding in intact HEK293-M_1_-cam5 cell monolayers. [^3^H]NMS saturation binding analysis determined a maximal binding (*B*_max_) value of 2.69±0.24 pmol mg^−1^ protein and a dissociation constant (*K*_D_) of 0.25±0.03 nM in HEK293-M_1_-cam5 cells (*n* = 4 independent experiments). HEK293-M_1_-cam5 cell monolayers were incubated with an approximate *K*_D_ value of [^3^H]NMS (∼0.3 nM) in the presence of varying agonist concentrations at 4°C for 4 h, or varying antagonist concentrations at 37°C for 45 min. The apparent binding affinities (p*K*_B_) for agonists and antagonists are summarized in Table 2. These data are comparable to affinity values obtained previously for the wild-type M_1_ mAChR receptor, e.g. see [27].

**Table 2:** Apparent binding affinities (expressed as −log *K*_B_ values) for various mAChR agonists and antagonists at the M_1_-cam5 AChR, determined by [^3^H]NMS competition binding.

	p*K*_B_	n
MCh	5.76±0.04	4
oxo-M	5.94±0.10	3
arecoline	5.03±0.03	3
AC-42	5.11±0.11	3
77-LH-28-1	6.17±0.12	3
atropine	8.67±0.09	5
pirenzepine	7.26±0.13	5

Data are shown as means ± s.e.m. for duplicate determinations in the number of separate experiments (n) indicated.

### Signalling and internalization properties of the M_1_-cam5 chimeric receptor

In order to determine if the chimeric receptor retained functional responses, accumulation of [^3^H]-inositol phosphates ([^3^H]IP_x_), ERK1/2 phosphorylation and receptor internalization have been monitored.

**Figure 3:**
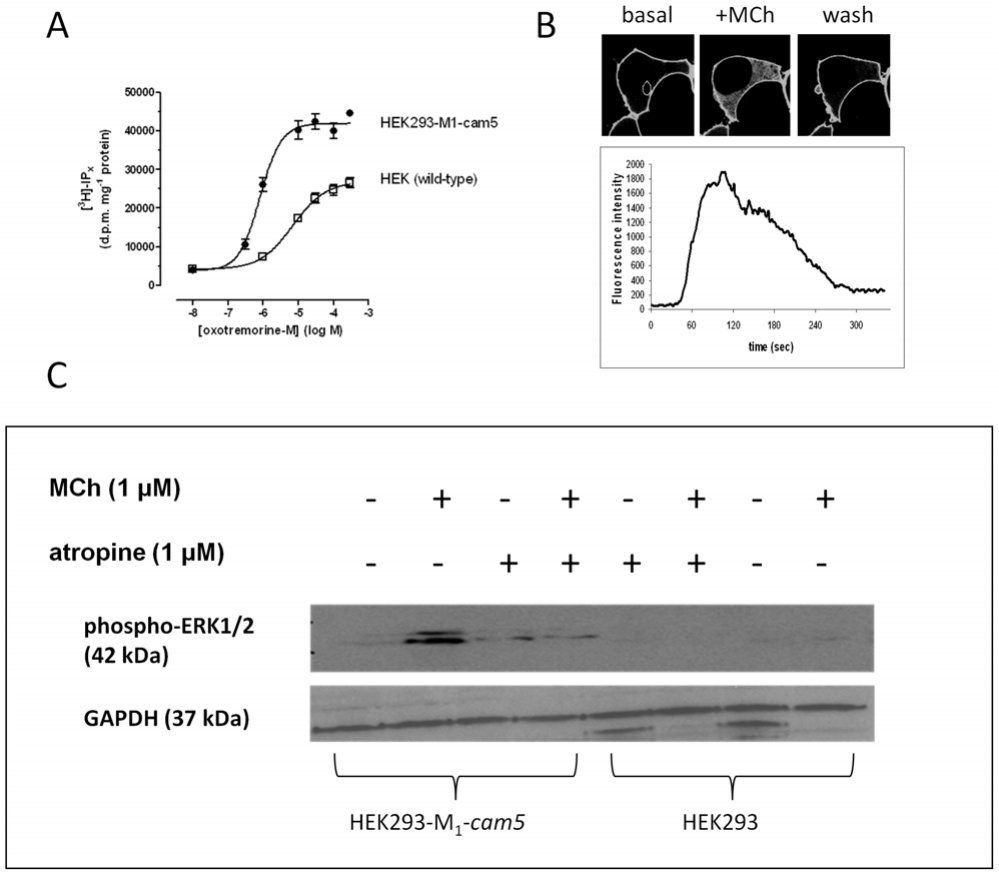
Signal transduction characteristics of M_1_-cam5 mAChR. **A**. Concentration-dependent [^3^H]IP_x_ accumulation in wild-type HEK293 (□) or HEK293-M1-cam5 (•) cells stimulated by oxo-M in the presence of 10 mM LiCl. **B**. Changes in phosphoinositide turnover (phosphatidylinositol 4,5-bisphosphate hydrolysis/Ins(1,4,5)P_3_ accumulation) in response to MCh in single HEK293-M_1_-cam5 cells using confocal fluorescence imaging. HEK293-M_1_-cam5 cells were transfected with the fluorescent biosensor eGFP-PH (see *Methods* section). Phosphoinositide turnover was assessed by monitoring the translocation of the eGFP-PH probe from the plasma membrane to the cytoplasm on addition of MCh (10 µM) for 60 s, followed by washout. The trace (B, lower panel) shows a representative time-course of change in cytoplasmic fluorescence intensity for 3–5 cells analyzed per coverslip over three separate experiments. **C**. MCh-induced activation of the ERK1/2 signalling cascade in HEK293-M_1_-cam5 cells. Cells were serum-starved for 24 h and then treated with MCh for 5 min in absence or presence of the mAChR antagonist atropine. A representative western blot is shown for phospho-ERK1/2 and GAPDH (loading control run in parallel) that was repeated independently two more times with similar results.

As an index of PLC activation, agonist-stimulated accumulation of total [^3^H]IP_x_ was assessed in the presence of Li^+^ (10 mM). Maximal stimulation with MCh caused an 11-fold increase in [^3^H]IP_x_ accumulation (41,954±1,030 d.p.m. mg^−1^ protein over a basal value of 3,781±1,792 d.p.m. mg^−1^ protein) with an EC_50_ of 0.8 µM (Fig. 3A). Wild-type HEK293 cells express M_3_ mAChRs (at approx. 50 fmol mg^−1^ protein) and MCh caused a smaller [^3^H]IP_x_ accumulation in these cells with an EC_50_>10 fold right-shifted relative to HEK293-M_1_-cam5 cells (Fig. 3A). Additionally, using confocal fluorescence imaging, we demonstrated that MCh (10 µM) was able to evoke a detectable translocation of the eGFP-PH biosensor from the plasma membrane to the cytoplasm in HEK293-M_1_-cam5 cells (but not wild-type HEK293 cells; data not shown), indicating the hydrolysis of phosphatidylinositol 4,5-bisphosphate and generation of IP_3_ by M_1_-cam5 (Fig. 3B). On removal of agonist, the eGFP-PH biosensor translocated back to the plasma membrane.

**Figure 4:**
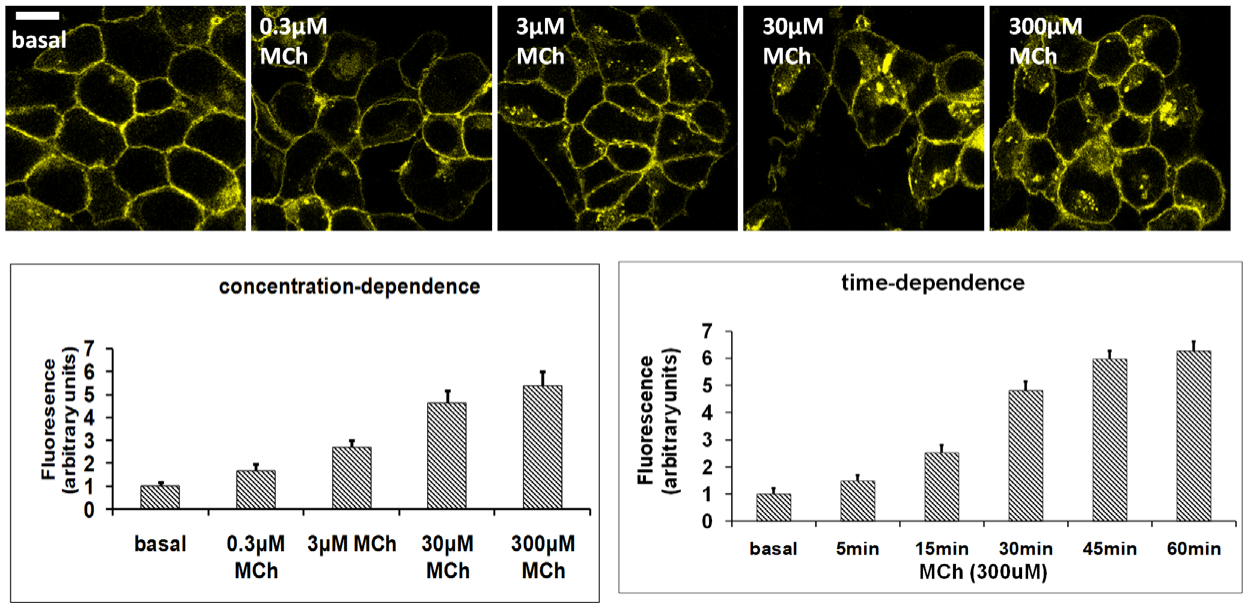
Internalization characteristics of the M_1_-cam5 mAChR stably expressed in HEK293 cells. HEK293-M_1_-cam5 cells were treated with various concentration of MCh for 45 min (to assess the concentration-dependency of receptor internalization), or with MCh (300 µM) for 0–60 min (to assess the time-dependency of receptor internalization). Cellular distributions of M_1_-cam5 mAChR were monitored by confocal microscopy. For quantification of intracellular fluorescence at least 10 individual cells in five random fields of view were examined as described in the Methods section. Data represent means ± s.e.m. from three independent experiments.

MCh treatment (1–300 µM for 5 min) of HEK293-M_1_-cam5 cells resulted in a rapid increase in ERK1/2 phosphorylation; an effect of agonist abolished in cell pre-incubated with atropine (1 µM; Fig. 3C). At relatively, low concentrations of MCh (1 µM; Fig. 3C) a robust increase in phospho-ERK1/2 was observed in HEK293-M_1_-cam5, but not wild-type HEK293 cells. Furthermore, the M_1_-cam5 mAChR internalized in response to MCh in a concentration- and time-dependent manner (Fig. 4), and recycled back to the plasma membrane on agonist removal (data not shown). Activation of the receptor with another full agonist, oxo-M, or the allosteric agonist AC-42 also resulted in receptor endocytosis (data not shown).

### Changes in FRET induced on ligand binding

**Figure 5:**
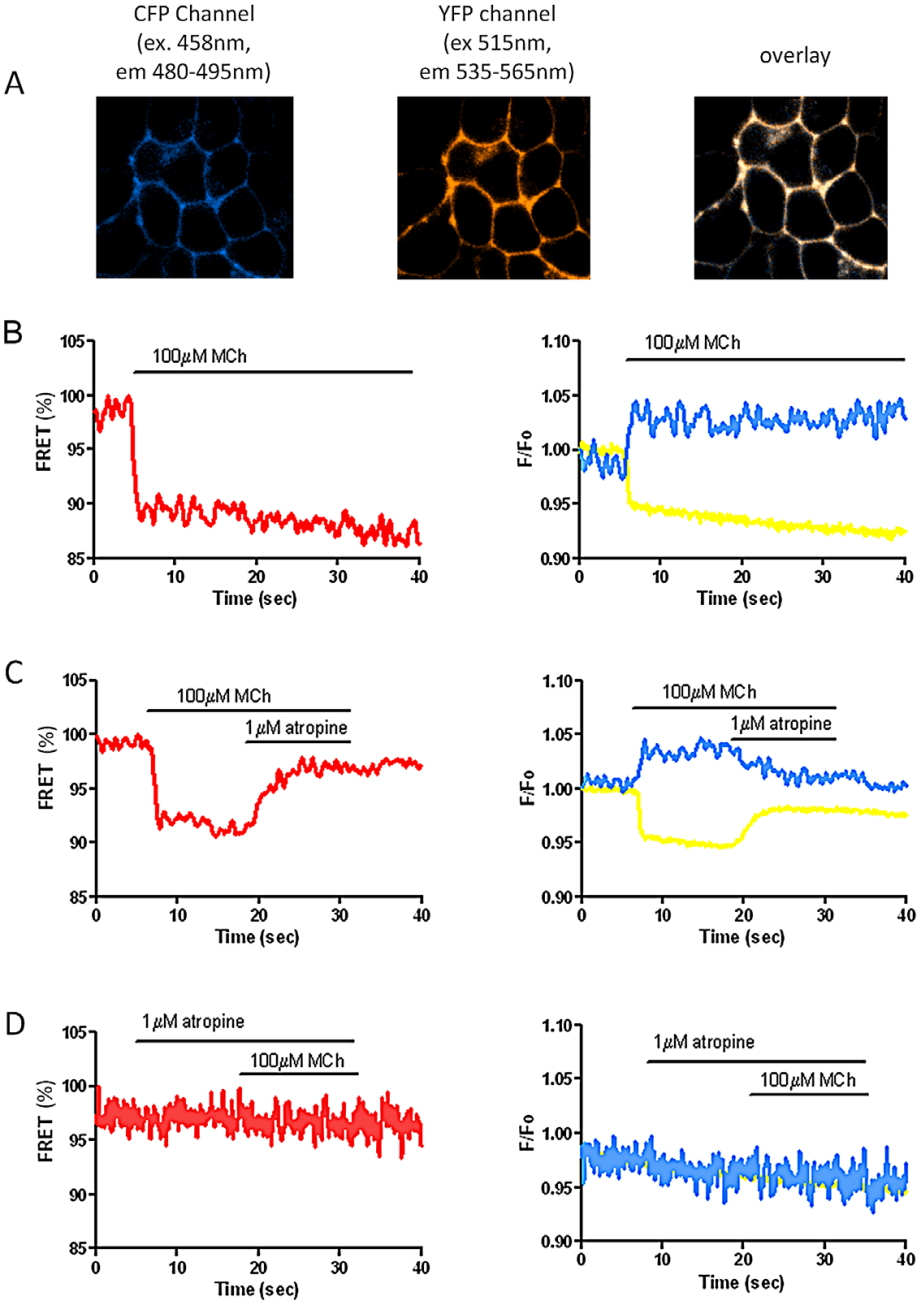
MCh-induced changes in FRET in HEK293 M_1_-cam5 cells. HEK293 cells stably expressing M_1_-cam5 were observed using fluorescence imaging with single wavelength excitation (436 nm) and dual wavelength emission (436 nm to detect ECFPc and 535 nm to detect EYFP^46L^. **A**. Representative images showing plasma membrane distribution of ECFPc (480 nm, left panel) and EYFP^46L^ (535 nm, middle panel), which overlap (right panel) as expected for signals from the same population of receptors. **B–D**, right panels: blue and yellow traces represent signals from ECFP and EYFP, respectively; left panels: red traces represent the FRET signal (ratio of F_EYFP_/F_ECFP_). Addition of MCh (100 µM) induced decreases in FRET, which remained constant throughout the application period (30–40 s; **B**); this effect was reversed on addition of atropine (1 µM; **C**); and the MCh-induced change in FRET ratio could be completely prevented by pre-addition of atropine (**D**). FRET data have been normalized so that the initial FRET signal is 100%. Emission traces are expressed as the change in fluorescence intensity from the basal fluorescence level (*F*/*F*_0_). Representative traces of at least three independent experiments are shown.

FRET was assessed using the ratio of normalized EYFP^F46L^/ECFPc fluorescence intensities. Addition of MCh (100 µM) leads to a rapid increase in ECFPc emission and decrease in EYFP^F46L^ emission, resulting in a reduction of FRET signal (Fig. 5B). Either washout of MCh or addition of atropine (1 µM) reversed the agonist-induced FRET change (Fig. 5C). The FRET change induced by MCh was completely prevented by pre-incubation with atropine (Fig. 5C). Control experiments with coexpression of M_1_-3ic-ECFPc and M_1_-cterm-EYFP^F46L^ in HEK293 cells showed no FRET response to MCh (300 µM; see Figure S1, Supporting Information). This indicates that the FRET signal detected from M_1_-cam5 mAChR results from intra-monomer conformational changes and not from intermolecular FRET in receptor dimers.

**Figure 6:**
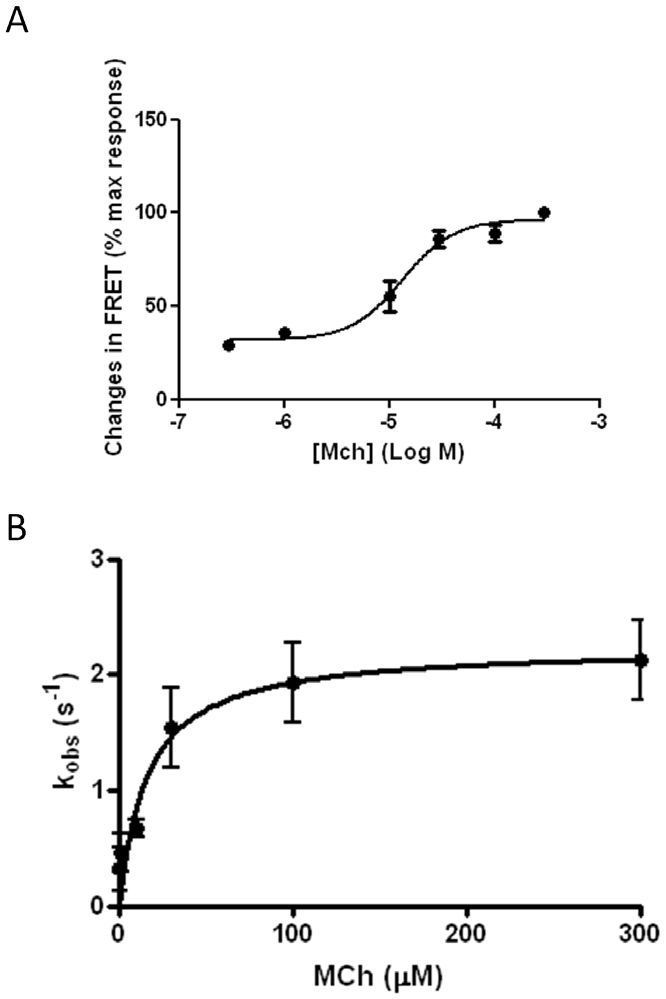
Concentration-dependency and dynamics of MCh-induced FRET changes in HEK293-M_1_-cam5 cells. **A**. HEK293 cells stably expressing M_1_-cam5 were stimulated with various concentration of MCh (0.3–300 µM) as above, and change in FRET ratio recorded. Data represent the means ± SEM from at least three independent experiments. **B**. Correlation between the rate constant (K*_obs_*) and MCh concentration was analysed as described in the Methods section. K*_obs_* values were obtained by fitting the FRET data to a single-phase exponential decay. Data represent the means ± s.e.m. from at least three independent experiments.

Stimulation of HEK293-M_1_-cam5 cells with increasing concentrations of MCh (0.3–300 µM; Fig. 6A) resulted in concentration-dependent changes in FRET, with a maximal FRET decrease of 9.8±0.4% induced at 300 µM MCh. We also assessed the kinetics of MCh-mediated receptor conformational change with time-resolved determinations of the FRET signal recorded from single cells on activation of M_1_-cam5 mAChR with various concentrations of MCh. Under all experimental conditions, the decrease in FRET signal followed a monophasic decay time-course, as described previously for the parathyroid hormone receptor and α_2A_-adrenoceptors [16]. As the concentration of MCh was increased, a faster time-course of FRET decrease was observed (Fig. 6B). The measured rate constant (K*_obs_*) increased across the MCh concentration range, reaching a maximum value at higher MCh concentrations (Fig. 6B).

**Figure 7:**
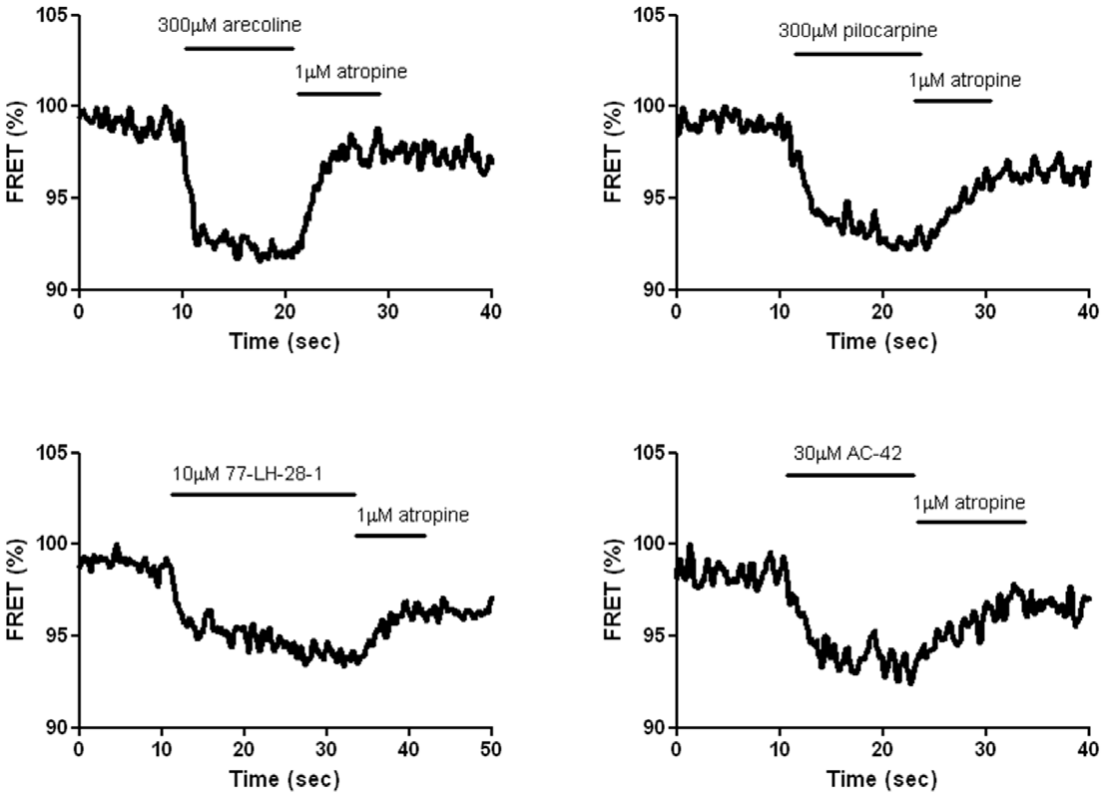
FRET changes induced by mAChR orthosteric and allosteric agonists in HEK293-M_1_-cam5 cells. Addition of arecoline (**A**; 300 µM), pilocarpine (**B**; 300 µM), 77-LH-28-1 (**C**; 10 µM) and AC-42 (**D**; 30 µM) induced decreases in FRET, which remained constant until reversal by addition of atropine (1 µM). FRET data have been normalized so that the initial FRET signal is 100%.

**Figure 8:**
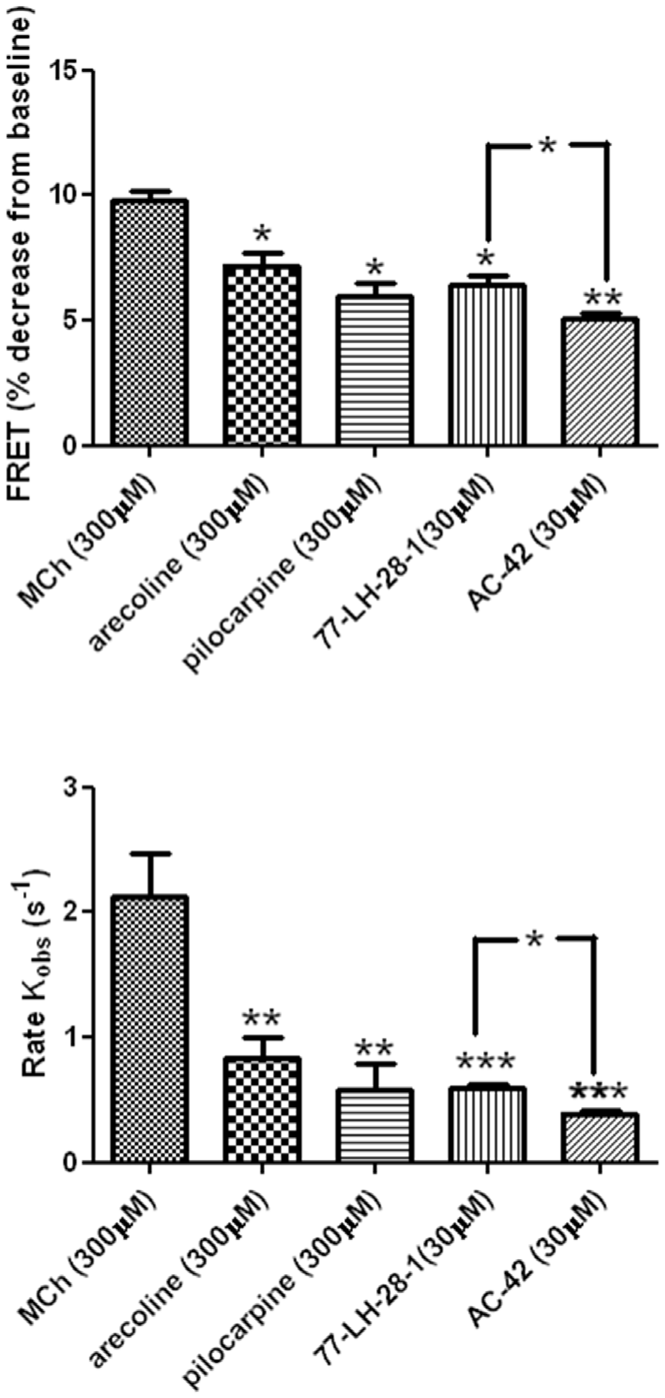
Comparisons of maximal FRET changes and rate constants for a variety of orthosteric and allosteric ligands in HEK293-M_1_-cam5 cells. Cells were stimulated with a maximally effective concentration of each agonist and FRET changes (**A**) and K*_obs_* values (**B**) were determined as described above. Data are presented as means ± s.e.m. from at least three independent experiments. One-way AVOVA (*p<0.05; **p<0.005; ***p<0.0001).

Next, we investigated the effects of various othosteric partial agonists (arecoline and pilocarpine) and allosteric agonists (AC-42 and 77-LH-28-1) on M_1_-cam5 mAChR FRET signals. As was found for MCh, all orthosteric/allosteric agonists caused reductions in intramolecular FRET, which were reversed on addition of atropine (1 µM; Fig. 7). Furthermore, pre-addition with either atropine (1 µM) or pirenzepine (10 µM) prior to agonist application prevented the FRET changes in all cases (data not shown). The full agonist, MCh, was however more effective in reducing FRET (9.8±0.4%) than arecoline (7.2±0.5%), pilocarpine (5.9±0.5%), 77-LH-28-1 (6.4±0.4%) and AC-42 (5.1±0.3%) (Fig. 8A). In addition, the rate constant (K*_obs_*) by which MCh induced a FRET decrease was more than two-fold greater than for any of other agonists tested (Fig. 8B).

## Discussion

In this study we report on a mouse M_1_ mAChR tagged with two genetically-encoded fluorescent proteins that allow real-time conformational changes in the receptor to be observed following activation by a number of ligands. The two fluorescent proteins, improved variants of CFP and YFP, were introduced into the i3 loop and the *C*-terminus, at positions in sufficient apposition for the unliganded receptor to generate a stable FRET signal. On binding of agonist, a decrease in FRET was observed, presumably generated through movement of the i3 domain relative to the *C*-terminus [10].

Both mouse and human M_1_ mAChRs have recently been modified by other groups to incorporate either ECFPc/EYFP [28], [29], or CFP/FlAsH (fluorescein arsenical hairpin) [30] pairs into i3/*C*-terminal domains. In the former example, ECFPc was introduced at the *C*-terminal and EYFP was inserted into the i3 loop replacing Ala223-Val358 of the wild-type receptor [29]. In the CFP/FlAsH chimeric M_1_ mAChR ECFP was introduced at the *C*-terminal, while the FlAsH motif (CCPGCC) was introduced into the i3 loop together with the amino acid sequence between Gly228 and Lys350 being deleted [30]. Thus, in contrast to our M_1_-cam5 construct, these other chimeras lack the flexible *C*-terminal linker sequence (LNSADI) and have differently located sequence insertions into the i3 domain together with substantial deletions from the wild-type M_1_ mAChR.

The location and/or full retention of the i3 domain in M_1_-cam5 resulted in the receptor exhibiting a full repertoire of cellular responses when stably expressed in HEK293 cells. Thus, on agonist addition phosphoinositide turnover, phosphorylation of ERK1/2 and receptor internalization could all be detected in HEK293-M_1_-cam5 cells suggesting that this chimeric receptor retains many of the signalling properties of the wild-type receptor. At present we do not know if, or to what extent, addition of ECFPc and/or EYFP^F46L^ compromises receptor function since we did not quantify the relative ability of M_1_-cam5 to couple to downstream signals compared to the untagged receptor. Nevertheless, our observations with M_1_-cam5 contrast with the findings of Jensen *et al.* [28] who reported that the ECFPc/EYFP mouse M_1_ mAChR was severely compromised with respect to downstream signalling, a commonly reported deficiency of CFP/YFP-GPCR chimeras [16], [31].

The maximum change in FRET observed in HEK293-M_1_-cam5 cells following addition of a full agonist was approximately 10%, which is similar or larger in size than the changes observed for CFP/YFP-based detectors incorporated into other family A GPCRs, including the A_2A_ adenosine (≈9%), B_2_ bradykinin (11%) α_2A_ adrenoceptor (≈6%), and M_2_ mAChR (≈6%) [31]–[34], but smaller than observed for the family B parathyroid hormone receptor (≈20%) [16]. The recently reported CFP/FlAsH chimeric M_1_ mAChR also exhibited a modest dynamic range of ≈7% [30], consistent with other reported CFP/FlAsH-GPCR chimeras [31].

An important application of GPCR intramolecular FRET has been to increase our understanding of the conformational changes that can occur following receptor binding of different classes of pharmacological ligand. For example, studies focusing on the α_2A_ adrenoceptor have provided evidence for conformational and kinetic differences when receptors are occupied by agonists, partial agonists and inverse agonists [16], [33], [35], [36]. We have shown that an M_1_ mAChR chimera (M_1_-cam5) can be activated by full (MCh) and partial orthosteric (pilocarpine and arecoline) and allosteric (AC-42 and 77-LH-28-1) agonists. Recent work from the Challiss laboratory investigated the intrinsic efficacies of this set of compounds using multiple readouts, including receptor-G protein-coupling, activation of phospholipase C and receptor desensitization/internalization [20], [27]. This work demonstrated that the allosteric agonists can stimulate M_1_-G_q/11_- and M_1_-G_s_-dependent signalling; but are less able to promote M_1_-G_i1/2_-coupling than otherwise equi-efficacious orthosteric agonists [20]. The present study complements this previous work by directly assessing drug-induced real-time conformational change in the M_1_-cam5 chimera. Orthosteric and allosteric partial agonists all caused significantly lower maximal changes in M_1_-cam5 FRET, which correlated well with previous rankings of these compounds based on efficacy readouts [20]. These data indicate that despite AC42 and 77-LH-28-1 binding at a site on the M_1_ mAChR distinct from the orthosterically-acting ligands [12], [14], [15], the kinetics and extent of conformational changes observed are indistinguishable from those evoked by equi-effective orthosteric partial agonists.

In addition, the rate of conformational change on agonist addition was significantly reduced (by >2-fold) for all partial agonists compared to the full orthosteric agonist, MCh (see Fig. 8). It should be noted that rate of conformational change (K*_obs_*) reported here on M_1_-cam5 binding to a full agonist is lower than values recently reported for other M_1_ mAChR chimeras [28], [30], and indeed other family A GPCRs, including the M_2_ mAChR [16], [34]. The precise reason for this difference is unclear, although it is known that the kinetics of agonist-induced conformational change is influenced by the location of the YFP/CFP reporter within the i3 loop [36] and by other factors including membrane fluidity and microenvironment [37]. In the case of M_1_-cam5, the chimera is stably expressed in HEK293 cells and does not contain the i3 deletions seen in other GPCR FRET constructs. Therefore, M_1_-cam5 is more likely to be trafficked to specific plasma membrane microenvironments (e.g. lipid rafts) than transiently expressed GPCRs and the rate of conformational change may be constrained by receptor-lipid and/or receptor protein interactions.

In addition to exploring the effects of orthosteric and allosteric agonist interactions with the M_1_-cam5 chimera, the effects of atropine and pirenzepine were also assessed. These compounds have been reported to possess inverse agonist activity at a number of mAChR subtypes [38]–[40]. A previous study clearly demonstrated FRET changes in a CFP/YFP-α_2A_ adrenoceptor chimera on addition of inverse agonists, such as yohimbine and rauwolscine. These changes were not only in the opposite direction to that caused by noradrenaline, and also displayed distinct kinetics [35]. While atropine and pirenzepine were able to both prevent and rapidly reverse orthosteric and allosteric agonist-stimulated FRET changes, addition of either agent alone had no effect on the basal M_1_-cam5 FRET signal. These data can be interpreted as atropine and pirenzepine lacking sufficient negative efficacy to cause a detectable1 change in basal M_1_-cam5 FRET, or more likely, the M_1_-cam5 lacks significant constitutive activity and resides in a ‘locked’, inactive state requiring agonist binding to undergo conformational change.

In conclusion, our data provide evidence that potency and efficacy differences among M_1_ mAChR orthosteric and allosteric agonists can be quantitatively assessed at the level of the receptor using the M_1_-cam5 chimeric receptor reported here. Despite the intramolecular incorporation of two ∼30 KDa proteins into the M_1_ mAChR structure the M_1_-cam5 chimera retains an ability to link to downstream signal transduction pathways and to traffic into intracellular compartments. This latter property may allow the construct to be used to observe receptor conformational changes that occur within organellar compartments of the cell during ongoing signalling and receptor processing towards either receptor resensitization or down-regulation.

## Supporting Information

Figure S1 **The agonist-evoked FRET responses of M1-cam5 do not result from movement of receptors within multimeric assemblies.** HEK-293 cells were transiently transfected with either (A) M1-cam5 or (B) two separate plasmids, one encoding M1 with a C-terminal YFP^F46L^ tag and the other encoding M1 with an ECFPc tag at the same third intracellular loop location as M1-cam5. ECFPc and EYFP^F46L^ fluorescence and percentage FRET changes were measured as described in the main methods section. MCh, methacholine. The traces are the average responses from 8 individual cells. In B, all cells used for analysis displayed robust ECFPc and EYFP^F46L^ fluorescence, thus indicating that both individually tagged M1 receptors were expressed. The traces are representative of responses from two separate transfections.
(TIF)
