# Supplementary figures and images for: FRET-Based Detection of M1 Muscarinic Acetylcholine Receptor Activation by Orthosteric and Allosteric Agonists
Source: PLoS One. 2012 Jan 17;7(1):e29946. doi: 10.1371/journal.pone.0029946 (PMC3260180; doi:10.1371/journal.pone.0029946)

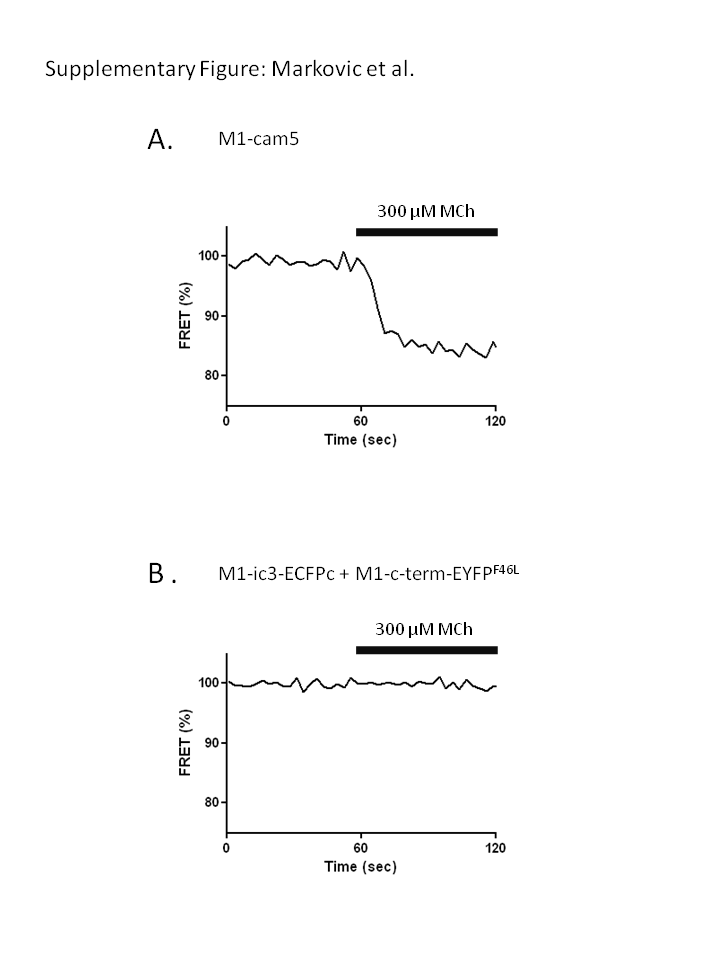

Supplement: Figure S1 — The agonist-evoked FRET responses of M1-cam5 do not result from movement of receptors within multimeric assemblies. HEK-293 cells were transiently transfected with either (A) M1-cam5 or (B) two separate plasmids, one encoding M1 with a C-terminal YFPF46L tag and the other encoding M1 with an ECFPc tag at the same third intracellular loop location as M1-cam5. ECFPc and EYFPF46L fluorescence and percentage FRET changes were measured as described in the main methods section. MCh, methacholine. The traces are the average responses from 8 individual cells. In B, all cells used for analysis displayed robust ECFPc and EYFPF46L fluorescence, thus indicating that both individually tagged M1 receptors were expressed. The traces are representative of responses from two separate transfections. (TIF) [file pone.0029946.s001.tif]
